# American Academy of Dental Science

**Published:** 1890-12

**Authors:** 

**Affiliations:** American Academy of Dental Science


### AMERICAN ACADEMY OF DENTAL SCIENCE.

THE American Academy of Dental Science held its regular monthly meeting May 7, 1890, in the Boston Medical Library Association rooms, President Seabury in the chair.

Dr. William P. Cooke read a paper upon "Formations in the Pulp-Cavity." (For Dr. Cooke's paper, see page 725.)

*Dr. Codman.*—I think this is too good a paper to pass without every one present saying something on it. Surely the facts are placed before us, and there were several which came up which seemed to me to be extremely interesting. The one that strikes me first, and is of the most interest to me, belongs to the sections of exostosed teeth. I was not aware before that exostosed teeth had this deposit as large or larger than others, and it brings up an interesting question in my mind,—Was the deposit in the pulp-cavity first, or the exostosis, or did they come together? It seems to me that the deposit in the pulp-cavity occurred first, though both may have taken place at the same time. It is a matter which, at first sight, does not seem to be of any great importance, but still it is a leading point towards the solving of the problem of the irritation. My theory is, and I believe it is also the theory of the essayist and most of the other gentlemen who have written upon this subject, that a certain amount of irritation produces these deposits. We have seen it illustrated by the fact that pulp-stones, at least in a great many of the cases, are deposited directly under fillings,—directly under cavities,—where the greatest amount of pulp-irritation has at some time taken place; hence, in endeavoring to solve the problem every point like this is worthy of consideration.

*Dr. Brackett.*—I must add my expression of appreciation and gratification for the paper that has been presented before us. I have been very much impressed with the improved method of demonstration over that prevailing in former times, and this, added to the foundation facts shown, has made it very interesting and instructive for us. It seems to me that the essential fact in connection with all of these cases, and that has not been very prominently mentioned, is the presence of an abundant supply of lime-salts in the system. I have never yet seen anything of this kind in a deciduous tooth, and I think it is the experience of most practitioners that the percentage of deposits of this nature increases with age, increases coincidently with the process of calcification in other tissues with advancing years, as it takes place in cartilage, as it takes place around the periphery of the pulp, as it takes place in the coats of the arteries and valves of the heart, and so on, indefinitely. I should simply add to the expression of the last speaker, suggesting irritation as a determining factor in the development of this condition, that we may assume as another element the abundant supply of lime-salts in the system. An analysis of the skeleton in the cases of teeth having this dense structure would, I think, show a similar result in nearly all its parts. That is to say, such teeth, having either of these conditions,—hypertrophy of the cementum, or calcification within the pulp-chamber,—are more apt to be found in persons having an abundant supply of lime-salts than in individuals where the supply of lime-salts is lacking.

*Dr. Eames.*—I am very much pleased to see the illustrations of this pathological condition. I have been struck with the force of the many facts that have been brought out, and shall easily remember them because they have been so plainly illustrated. I am moved to speak because I feel that there are those who have noticed these formations early in life. I must say that, so far as I have observed, I have seen more of them existing in persons in early life, even in deciduous teeth, but more particularly in young persons of from twelve to eighteen years of age. It has been pretty well shown that these deposits originate from irritation, and there may be a variety of these conditions, and not so much because there is an abundant supply of lime-salts in the system. For some reason the lime-salts may be diverted from their regular channels and be deposited here or there. We may fill the system, saturate it, so to speak, with lime-salts, and if the natural channels, with their corresponding nerve-supply, are in a healthy condition, the lime-salts will not be found in excess anywhere in the body, neither on the skeleton, nor in the pulp-cavity, nor on the teeth in the shape of exostosis. There are lime-salts enough in the common food that we eat daily to supply what is needed for a normal condition; but even if we take a case that is not plentifully supplied with lime-salts and produce a sufficient irritation, we may have an irregular formation in the system, an exostosis, because of the irritant. The fact that we find these formations in adults does not prove the time of their origin. They may have begun in childhood and kept increasing more or less slowly until they come to our notice later in life.

I might say that within two or three weeks I amputated a tooth which was apparently sound. There was certainly no pain or discomfort to the patient, and I found quite a large ossified condition in the pulp. It was a right superior lateral.

Dr. H. F. Hamilton then read a paper on "The Combination of Tin and Gold as a Filling-Material." (For Dr. Hamilton's paper, see page 738.)

*Dr. Briggs.*—I would like to add my testimony to that of Dr. Hamilton with regard to this combination. Dr. Hamilton spoke to me about it some two and a half years ago. I tried it in several cases and waited a year to see what the result would be. In the mean time I did not hear anything further about it, and did not know of any one else using it, but at the end of a year the results were so satisfactory that I have been using it ever since. There is scarcely a day passes that I do not put in one or more of these fillings. I use it in many of those cases where formerly, as Dr. Hamilton says, I used cement or gutta-percha. I feel it is almost as conservative a filling as either, and almost as easy to put in, while it is of course perfectly durable.

*Dr. Williams.*—I should think it was about six years ago that a lady came to me who had been a patient of Dr. Frank Abbott, in Berlin, and showed me some fillings of this combination, which she stated Dr. Abbott wished me particularly to inspect. He also sent a letter to me which she had lost, so that I was unable to know what he had to say about them. I found them doing admirably, several of them being large fillings, and all of them in approximal cavities, up near the borders of the gum, well preserved. I kept watch of that case, but I found that the inveterate enemy caries did get in at one or two places. It is not a specific; it does not prevent decay absolutely.

*Dr. Banfield.*—I would like to ask Dr. Hamilton if the method requires considerable separation before insertion?

*Dr. Hamilton.*—With this, as with other materials, space is an advantage. I separate about one-half as much as for a filling of gold alone.

*Dr. Clapp.*—What gold do you use?

*Dr. Hamilton.*—I use Knapp's foil. It is non-cohesive and softer than soft gold.

*Dr. Taft.*—This subject of combination fillings has long been an interesting one to me, and though I have had no experience as yet with this material that Dr. Hamilton speaks of, I feel encouraged to try it after listening to his paper, and I am very glad he has brought the subject before us. The article in the paper to which he has referred I do not remember having seen, but I shall take a good deal of interest in reading it over. I should like to ask if the filling is put in exactly like a soft gold filling, that is to say, by the wedge process?

*Dr. Hamilton.*—Yes, as nearly as my experience with soft gold is concerned. I have never used very much soft gold.

*Dr. Codman.*—I think most of us who have read the *Dental Cosmos* must have noticed the articles on combination fillings that have appeared from time to time, but a good many of us have not tried them, because there seems to be no reason why the combination of two *pure* metals, as tin and gold are supposed to be, should produce any new result. Now we would, all of us, like to know, if it is really so, if there is any change which goes on between these two metals. I do not pretend to be up in chemistry to know for a certainty, but it seems to me that no change can take place unless there is an intervention of a third oxidizable metal. The ordinary tin-foil is not pure, it contains lead, and it is possible that the combination of the tin, the gold, and the lead produces the result spoken of. When the metals are brought into contact the lead begins to oxidize at once, and this enlarges the filling by a slight increase of size. It is tight, and grows tighter by the very fact of its enlargement. This may be the philosophy of it. Of course, there is still pure metal enough to hold the filling together and hold it strongly, but there seems to be some reason why the combination of two metals should be better adapted than either of the metals individually.

*Dr. Williams.*—Does Dr. Codman think that the tin-foil that is usually sold contains lead to any appreciable extent?

*Dr. Codman.*—Yes, sir. In chemistry it is not always necessary that there should be a large amount of the intervening metal to bring about action between the other two metals. I really do not believe that there is lead enough in the tin-foil to do any physical harm, but I do believe that a large proportion of it contains some lead.

PRESENTATION OF SPECIMENS.

*Dr. Smith.*—I received a letter from Dr. Brackett this afternoon, saying that he would be present,—possibly late,—and would have with him a warm-air apparatus, as he expresses it. I did not receive it in time to have it put on the card. Also, I did not know of Dr. Hamilton's paper in time to have it announced, but Dr. Hamilton has had a standing invitation to read a paper whenever he feels so inclined.

*Dr. Brackett.*—Mr. President and gentlemen, this is an apparatus for which I am indebted to our friend Dr. Bogue, of New York, and it came to my mind, just as I was starting from home, that I was indebted to him for a great many things, tangible and intangible. Of the tangible things, I have here one of the earlier forms of apparatus for pressing teeth apart immediately. Of all such contrivances, it is the one which I use with the most satisfaction. It is especially applicable to molars and bicuspids, and is one of two instruments which he made; one he kept himself, and this one he gave to me.

This is one of a pair of instruments that are partially home-made by Dr. Bogue, and is presented simply as a mirror, the narrow oval of which makes it especially applicable in reaching far back in a mouth that does not open very wide. The glass and frame were made by C. Ash & Sons; the handle is an individual thing.

The warm-air apparatus is designed especially for lessening the sensitiveness of cavities. Of the variety of principles upon which obtundents operate, one of the most valuable is the principle of dryness. If we imagine, for the sake of explanation, a jelly-fish completely desiccated, and compare it with the jelly-fish under normal conditions, a marked difference in everything that goes to make up the functions of its tissues would be apparent. Applying this principle, we can easily understand the difference between the cutting of a tooth when it is freshly extracted and cutting it after it has been exposed to dry air for indefinite months. It seems to me that of all means for obtaining dryness by a hot blast this apparatus is as simple as anything can be. The cylinder is heated over an alcohol lamp, or Bunsen burner, and air is supplied to it by means of a simple rubber bulb, with valves, and an interposed elastic chamber, the whole similar in operation to the blower used in soldering. The amount of heat can be regulated by the amount of heat that is given to the cylinder, or by the rapidity of blowing and the distance that it is held away from the tooth. In those cases where dryness can be applied I have never used anything so satisfactory, and apparently so harmless, as this very simple apparatus. It is the design of a French workman, not a dentist, and I believe they are not yet to be had in this country. Just what they will cost I am unable to say, but in France, I believe, the cost is about six dollars. The success which Dr. Shumway had in making cohesive gold work satisfactorily with the smooth instruments which he used was directly dependent upon the attainment of great dryness. You will find, by holding that close to the face, quite a little degree of heat, and the circumstance of the current of air steadily flowing does a very great deal in accomplishing dryness. It is possible in a few minutes to make a tooth cut so that it will ring under the excavator like a tooth that has been extracted for several months. In exhibiting this apparatus before the Academy I do not wish to take any of the honor to myself, but I present it to you as the contribution of our friend Dr. Bogue.

There is one point in connection with the warm-air apparatus which I forgot to mention, and that is, it is a very nice help to hasten the hardening of a cement filling, thereby saving time, and adding to the durability of the filling.

WILLIAM H. POTTER, D.M.D.,
*Editor American Academy of Dental Science.*

